# Patient Satisfaction and Outcomes of Penile Prosthesis Implantation in Psychogenic and Organic Erectile Dysfunction: A Comparative Study

**DOI:** 10.3390/jcm14145032

**Published:** 2025-07-16

**Authors:** Maurizio De Rocco Ponce, Alejandro Silva Garretón, Ángela Sousa Iglesias, Sebastian Dumas Castro, Ricardo Contreras Garcia, Luis Malca Caballero, Josvany Rene Sanchez Curbelo, Doron Vantman Luft, Eduard Ruiz Castañé, Osvaldo Rajmil

**Affiliations:** 1 Andrology Department, Fundació Puigvert, Instituto de Investigaciones Biomédicas Sant Pau (IIB-Sant Pau), Universitat Autònoma de Barcelona, 08041 Barcelona, Spain; sdumas@fundacio-puigvert.es (S.D.C.); rcontreras@fundacio-puigvert.es (R.C.G.); lmalca@fundacio-puigvert.es (L.M.C.); jrsanchez@fundacio-puigvert.es (J.R.S.C.); doronv@gmail.com (D.V.L.); eruiz@fudacio-puigvert.es (E.R.C.); orajmil@fundacio-puigvert.es (O.R.); 2 Departament de Medicina, Universitat Autònoma de Barcelona, 08041 Barcelona, Spain; 3 UROINTEC, 35012 Las Palmas de Gran Canaria, Spain; drsilvagarreton@gmail.com; 4 Servicio de Uroloxía, Complexo Hospitalario Universitario de Pontevedra, 36071 Pontevedra, Spain; angela150290@gmail.com

**Keywords:** penile prosthesis, erectile dysfunction, patient satisfaction, Psychogenic Sexual Dysfunctions, postoperative complications

## Abstract

**Background**: Penile prosthesis implantation (PPI) is an established treatment for erectile dysfunction (ED). Nevertheless, the effectiveness of and satisfaction with PPI in mainly psychogenic ED compared to mainly organic ED patients remain underexplored. **Aim**: To evaluate patient satisfaction outcomes following PPI in individuals diagnosed as mainly psychogenic ED vs. mainly organic ED. **Methods**: Twenty-five patients with psychogenic ED who underwent PPI were included. Data were collected from medical records and a follow-up assessment was done using the Quality of Life and Sexuality with Penile Prosthesis (QolSPP) questionnaire. Additionally, the patients filled out an ad hoc questionnaire including self-reported satisfaction rated on a 1-to-10 scale, the Global Assessment Questionnaire-Questions 1 and 2 (GAQ-1, 2), and the Sexual Encounter Profile Questions 2 and 5 (SEP-2, 5). Results were compared with those of 36 patients with mainly organic ED (control) for comparative analysis. **Results**: In the psychogenic ED group, 96% reported improved erections, 92% felt more confident initiating sex, 92% achieved penetration and 95% had satisfactory sexual encounters. The overall satisfaction score was 8.71 on a 10-point scale. Comparative analysis using the QolSPP questionnaire revealed statistically significant differences favouring the psychogenic group in 8 of 16 questions, regarding prosthesis satisfaction and overall well-being. Surgical complications were noted in 16% of the psychogenic group, compared to a 2.8% complication rate in the organic ED control group. **Conclusions**: The findings indicate high levels of satisfaction with PPI among patients with psychogenic ED, comparable to those with organic ED. However, an increase in complications in the psychogenic cohort highlights the need for careful consideration of surgical risks in this population.

## 1. Introduction

According to the World Health Organisation [1], sexual health is defined as “a state of physical, mental and social well-being in relation to sexuality.”

Erectile Dysfunction (ED) is defined as the persistent inability to achieve and/or maintain an erection rigid enough for satisfactory sexual intercourse. This has implications not only in organic aspects but also affects the psychosexual sphere of the individual, which can significantly impact the patient from a psychosocial perspective and influence both their quality of life and that of their partner [2,3].

The global prevalence of this pathology according to the Massachusetts Male Aging Study (MMAS) is 52% in patients between 40 and 70 years old, with 17.2% mild forms, 25.2% moderate forms and 9.6% severe forms [4].

The pathophysiology of ED involves multiple mechanisms including vasculogenic, neurogenic, endocrinological, anatomical, pharmacological, traumatic and psychogenic causes, with several of them coexisting in the same patient in most cases [5]. Among the previously mentioned factors, the most important are age, cardiovascular disease, high blood pressure, dyslipidaemia, smoking, diabetes mellitus, obesity and sedentary lifestyle [6,7].

In fact, in men under 40 years old, around 83% of cases have a predominantly psychogenic cause, while around 60% of those over 40 years have an organic cause as the most likely aetiology, with a mixed origin being very common [8,9]. In this context, a proper diagnosis work-up is mandatory, which can be expected to lead to the best approach and more effective treatment.

The diagnosis begins with a detailed clinical history and physical examination [10]. The anamnesis should include the medical history of the patient and his partner, as well as their sexual history [11]. Validated questionnaires are a useful tool: the IIEF (International Index for Erectile Dysfunction) is one of the most globally used and is focused on different sexual function domains (erectile orgasmic functions, sexual desire, satisfaction with the sexual act and global satisfaction). It is also widely used to measure the results of the treatments implemented [12,13].

Complementary studies are often required for a complete diagnosis. They include general and hormonal blood analyses [14] and second-line tests such as penile colour Doppler ultrasound (PCDU) [15,16] and/or the nocturnal penile rigidity and tumescence test (NPTR) [17].

The treatment of ED should begin with patient education on lifestyle changes and modifiable risk factors, such as sedentarism, smoking, obesity or evaluation of usual medication, among others [18,19,20,21]. Oral medication with phosphodiesterase 5 inhibitors (PDE5 inhibitors) is the first-line treatment, which can be utilised after or during the aforementioned interventions [22,23].

The second-line treatment options are intraurethral alprostadil, intracavernous injection of vasoactive drugs and vacuum devices [24,25,26,27,28].

The third-line treatment option for cases of ED that are resistant to less invasive therapies is the surgical implantation of a penile prosthesis (PPI) [29], which is widely accepted to treat severe organic ED. On the other hand, in cases of severe psychogenic ED that do not respond to medical treatments, the recommendation for PPI remains a topic of debate.

A high patient satisfaction rate (around 80%) has been reported for PPI. Nevertheless, the publications regarding satisfaction after implantation have focused on patients with a generic ED, without making any etiological classification [30,31]. On the other hand, the data published to date regarding the satisfaction of psychogenic ED patients after penile prosthesis implantation is scarce.

The aim of the present study is to assess the clinical outcomes of PPI in patients with mainly psychogenic ED.

## 2. Material and Methods

Type of study. The present work is a retrospective case-control study conducted in a single tertiary referral centre.

Outcomes. Primary outcome: to assess the satisfaction rate after PPI in patients with a diagnosis of mainly psychogenic ED. Secondary outcomes: (1) satisfaction rate and (2) rate of surgical complications after PPI between patients with mainly psychogenic ED vs. patients with mainly organic ED (controls).

Inclusion and exclusion criteria. All cases and controls were patients consecutively included who underwent inflatable penile prosthesis implantation to treat ED which was refractory to first- and second-line medical treatments. Case-patients group criterion: men with a diagnosis of ED with a dominant psychogenic cause. Control-patients group criterion: men with ED of a dominant organic cause.

The diagnosis of ED of mainly psychogenic or mainly organic cause in these patients was based on the clinical presentation, a psychological assessment by an experienced sexologist and the result of the nocturnal penile tumescence and rigidity test (NPTR, Rigiscan^®^). A 3-night NPTR test showing at least one erection with rigidity of 60% for 10 min or more was considered normal [32,33].

Patients with Peyronie’s disease, hypogonadism or previous PPI (non-naïve patients) were excluded.

Before the study, all enrolled patients signed an informed consent that received approval from the local Investigation Research Bureau (code C2023/25, 14 March 2024).

Protocol. All patients underwent PPI with an inflatable device: the Coloplast Titan Touch™ Inflatable Penile Prosthesis (Coloplast Corp., Minneapolis, MN, USA) or the AMS 700™ Inflatable Penile Prosthesis (Boston Scientific, Marlborough, MA, USA). All patients were previously treated with a preoperative prophylactic antibiotic dose of cefazolin 2g. The PPI was performed by the same surgical team via a peno-scrotal access. Patients were evaluated with a follow-up of at least 6 months after PPI using the QoLSPP (Quality of Life and Sexuality with Penile Prosthesis) questionnaire proposed by Caraceni et al. (Annex 1). This questionnaire is organised into four main domains: functional (referred to prosthesis function), personal, relational, and social. Each item within these domains is rated on a scale from 0 to 5, with higher numbers indicating greater satisfaction or a more positive experience. A response of 3 or above is generally interpreted as a positive outcome. The questionnaire is scored by summing the responses within each domain, leading to a domain-specific score. The functional domain can contribute up to 25 points, the personal domain up to 15 points, the relational domain up to 20 points, and the social domain up to 15 points. The total score, which is the sum of all domain scores, reflects the overall quality of life and satisfaction with the penile prosthesis.

Patients with mainly psychogenic ED (considered the case group) also answered an ad hoc questionnaire specifically formulated for the present work (Annex 2). The ad hoc questionnaire collected different data regarding their sex life after PPI. It included self-reported subjective satisfaction on a 1-to-10 scale, the Global Assessment Questionnaire-Questions 1 and 2 (GAQ-1: Has the treatment you have been taking improved your erectile function?; GAQ-2: If yes, has the treatment improved your ability to engage in sexual activity?), and the Sexual Encounter Profile Questions 2 and 5 (SEP-2: Were you able to insert your penis into your partner’s vagina?; SEP-5: Were you satisfied with the overall sexual experience?). Moreover, the ad hoc questionnaire also enquired about the satisfaction of the partner (as reported by the patient), the patients’ satisfaction from an “aesthetic” point of view and the actual use of the prosthesis after surgery, among other items.

Statistical analysis. Statistical analysis was performed using the SPSS statistical software for Windows (Version 23, SPSS Inc., Chicago, IL, USA). The Kolmogorov–Smirnov test was used to test for normal distribution; as not all variables presented a normal distribution, we used non-parametric methods. Continuous variables are expressed as median and 25–75 percentile interquartile range. Comparisons between subgroups were performed with the Wilcoxon–Mann–Whitney test. Categorical variables are expressed as frequencies and percentages, and were compared between groups using Pearson’s chi-square test. All reported probability values are two-tailed and a *p*-value < 0.05 was considered statistically significant.

## 3. Results

A total of 61 patients were enrolled, of which 25 were included in the case group (mainly psychogenic ED, PSY) and 36 patients were included in the control group (mainly organic ED, ORG). The whole sample had a median age of 63 (57–66) years, with a median duration of ED of 6 years. Among the present risk factors, 13 (21%) were smokers, 35 (58%) had hypertension, 24 (40%) had diabetes mellitus, while 41 (67%) had dyslipidaemia. Among our patients, 15 (25%) had a diagnosed coronary artery disease. Comparing the two sub-groups (PSY vs. ORG), there were no significant differences except in the prevalence of smokers and hypertension (both higher in the PSY group). The main characteristics of the study groups are summarised in Table 1.

The analysis of the 25 PSY patients is detailed below. The subjective satisfaction reported by the patients on a scale of 1 to 10 was 8.7/10, with a range of 5 to 10 points. Only one patient (4%) reported being unsatisfied (score 1/10), while the remaining 24 patients (96%) affirmed that the prothesis met their expectations. The patient who was not satisfied said that the prosthesis did not provide sufficient rigidity and caused pain during use. On the other hand, the other 24 patients reported that the penile prosthesis implant improved their erections (GAQ-1 question). Of these, 22 patients (92%) also believed that the implant improved their confidence to attempt to initiate a sexual relationship (GAQ-2 question), while 1 patient said not feeling the same with the penis as before and another mentioned noticing penile shortening.

Regarding whether the patient was able to penetrate their partner (SEP-2 question), 23 patients (92%) reported being able to penetrate. Of the 2 patients (8%) who reported being unable to penetrate, 1 mentioned failing due to shorter penile length and pain when activating the prosthesis, and the other mentioned not being able to establish sexual relationships with anyone after the prosthesis placement.

The SEP-5 question is about overall sexual satisfaction. Of the 23 patients who answered affirmatively to the SEP-2 question (ability to penetrate), 22 patients (95%) also responded affirmatively to the SEP-5 question. The only patient (5%) who said he did not have a satisfactory sexual relationship mentioned that his partner had lubrication and pain problems during penetration due to gynaecological causes, which ultimately prevented him from enjoying the sexual intercourse.

The partner’s satisfaction (as reported by the patient) was another item of the questionnaires. All PSY patients reported having only heterosexual relationships, 20 of them (80%) had a stable sexual partner and, of these, 14 (70%) had a sexually active partner—meaning that they actively sought sexual relations within the partnership. Among the 20 patients with a stable sexual partner, 16 of them (80%) reported that their partner was satisfied with the decision to have a penile prosthesis implanted. Of the four patients (20%) who reported that their partners were not satisfied with the penile prosthesis, one complained about insufficient rigidity and pain during activation, one mentioned female sexual dysfunction issues, another reported some discomfort with the prosthesis tubes in the scrotum and the tip of the cylinders during penetration, and the last one mentioned that his partner found it “excessively rigid.”

Regarding the patients’ satisfaction with the “aesthetic” result, 6 (24%) reported no noticeable change in the shape of the penis after the prosthesis placement, while 19 (76%) reported some morphological change in their penis: 14 (74%) described a shorter penile length, 4 (21%) a smaller girth and 1 (5%) some penile curvature.

Regarding the actual use of the prosthesis after surgery, 4 patients (16%) reported not having used it. Of the 21 patients who did use the prosthesis, only 4 patients (19%) reported having more than 10 sexual encounters per month, while the remaining 17 patients (81%) reported having fewer than 10 sexual encounters per month, with an average of almost 4 (3.88) sexual encounters per month.

Finally, we asked our PSY patients if they would get a penile prosthesis again. Twenty-one of them (88%) answered yes, while three of them (12%) answered no. Of the latter, one mentioned insufficient rigidity and pain during activation, and the other two mentioned postoperative discomfort—both physically and in terms of the “psychological stress” of surgery. Meanwhile, all those who would get it again responded that they would recommend this therapeutic modality and, of the three who would not get it again, one would recommend it because he understood that the postoperative experience would not necessarily be the same for all patients.

The comparative analysis with the control group was based on the QoLSPP questionnaire data. We observed statistically significant differences between PSY and ORG in 8 of the 16 questions. Specifically, in questions 3, 6, 7, 8, 10, 13, 14 and 15 we observed significantly higher scores in the PSY group. Questions 3, 6 and 7 pertain to the functional domain; question 8 to the relational domain; question 10 to the social domain; and questions 13, 14 and 15 to the personal domain (self-image). The results of this comparative analysis are shown in Table 2.

We compared the rate of surgical complications—both minor and major—in the two groups of patients. Among the PSY cases, there were 2 with surgical complications, accounting for 8% of this group. One of them had a wound dehiscence and hematoma, and the other reported a prosthesis malfunction requiring surgical revision (without prosthesis replacement). In the ORG group, only 1 patient (2.8%) reported a complication—which was minor, consisting of ecchymosis—without statistical significance (*p* = 0.860).

## 4. Discussion

This study aims to evaluate the satisfaction achieved through penile prosthesis implantation in patients with psychogenic ED and, additionally, to investigate whether there are differences in surgical outcomes in comparison with those with organic ED. This latter aspect is crucial for addressing the clinical question of whether patients with refractory psychogenic ED may have the same indication for penile prosthesis implantation as those with organic ED. We could not find significant literature on this topic. Perhaps the only example is the work of Schlamowitz et al. [34] in 1982, in which 17 patients and their sexual partners were included. They found somewhat less satisfaction and more postoperative complications in psychogenic patients. However, this investigation was done with few patients and more than 40 years ago, with different surgical techniques and prosthetic material from those we use today.

Assessing satisfaction with penile prosthesis implantation is complex, as it involves multiple aspects of a person’s life. In 2020, Barton et al. reviewed the literature from 1989 to 2018 related to this treatment and sexual quality of life, finding that 85% of patients were satisfied and that quality of life improved substantially for both the patient and their partner [35]. In fact, most studies on satisfaction repeatedly show patient and partner satisfaction rates around 80–90%, although with varying measurement tools, highlighting the need for a single validated tool that allows for reliable comparison of results [36,37].

In 2021, Manfredi et al. conducted a review of current evidence on patient and partner satisfaction with penile prosthesis implantation and showed that, although satisfaction rates exceed 90%, not all published studies used validated tools and existing questionnaires; moreover, many did not assess whether patient expectations were met and did not differentiate the results based on the cause of ED [38].

We found a 96% overall satisfaction among PSY patients and an average score of 8.71 on a 1–10 visual analogue scale (VAS). This result is comparable to what is found in the literature. Moreover, 88% of our patients would undergo the procedure again and would recommend it to others with the same problem. Those who would not undergo the procedure again cited insufficient rigidity, pain with activation or perioperative discomfort (both physical and psychological) as reasons. This, once again, demonstrates that understanding these factors is crucial when assessing the indication for surgery. Previous studies have indicated that key factors influencing patient satisfaction include enhanced erectile function, psychosocial benefits (such as increased self-esteem, self-confidence and positive emotions) and improved partner relationships. On the other hand, the main reported reasons for dissatisfaction are unrealistic expectations, reduced penile length, “unnatural” erections, malfunction of the prosthesis or issues related to the partner. Croce et al. also found that patients with Peyronie’s disease benefit the most from prosthesis implantation in terms of overall satisfaction; however, we cannot provide new data in this regard as we excluded patients with Peyronie’s diseases from the study.

One of the most reported unrealistic expectations in the literature is the belief that the penile prosthesis will add penile length, with some patients complaining of penile shortening after implantation. Palasi et al. published an article in 2022 questioning whether knowing the preoperative penile length influences patient satisfaction after prosthesis implantation, showing that measuring penile length before and after implantation does not change perceptions of satisfaction with the treatment, underscoring the need for preoperative expectation counselling [39].

Another interesting finding in the present study is the high partner satisfaction (80% among patients with a stable sexual partner). Partners who were not completely satisfied reported pain with penetration, discomfort with the hydraulic prosthesis tubes and untreated female sexual dysfunction issues. Remarkably, despite high satisfaction levels, the disparity between patients and their partners highlights the need for a thorough assessment of factors that may affect each partner’s satisfaction with the treatment.

To compare the satisfaction rates between patients with psychogenic ED and controls with organic ED, we used the questionnaire proposed by Caraceni et al. [40] which explores different domains as described in the Material and Methods. Statistically significant differences favouring the PSY group were observed for 8 of 16 questions. In the function domain, the differences regarding satisfaction and expectations met with the prosthesis were more pronounced, although the reason for this remains unclear. In the relational domain (i.e., partner relationship), only the question regarding partner satisfaction with prosthesis function showed a difference; however, globally it seems that partners of both groups did not present major differences, possibly because sexual satisfaction is based on more variables than just penile rigidity. In the social domain (relationship with the external world), differences emerged in only one question related to life satisfaction. This is interesting, as it suggests that this group of patients may have a particularly focused view on how they achieve life satisfaction, with sexuality serving as a fundamental pillar. This perspective could provide a psychological explanation for their ED, making it an intriguing area for future research. In the personal domain (self-image), we found that psychogenic patients reported feeling “more alive,” having more sexual desire and feeling more confident about engaging in sexual activity when compared to organic patients—areas that likely reflect aspects of patient self-esteem, which are highly marked in psychogenic patients. These findings suggest that functional domains and self-image are fundamental components for psychogenic patients. However, analysing the questions within each domain in detail could reveal important elements of the patient’s sexual history, potentially uncovering factors contributing to their ED.

Regarding surgical complications, we found a prosthetic infection rate of 4% in PSY patients (1 Clavien–Dindo II and 1 Clavien-Dindo IIIb complication) [41], which is comparable to the reported rate in the literature (1.5–3%) [42]. Moreover, PSY patients had a higher overall complication rate (8%) compared to the organic group (2.8%); however, this difference was not statistically significant.

The strengths of the present study include conducting a comparative analysis with a control group and using a specific questionnaire. On the other hand, it has some limitations: the sample size is relatively small, and we had to translate the QoLSPP into Spanish (as it has only been published in English and validated in Italian), which may have affected the accuracy of data collection. Moreover, in recent years, contemporary classifications of erectile dysfunction (ED) have moved beyond the traditional dichotomy of “organic” versus “psychogenic” causes. The 11th revision of the International Classification of Diseases (ICD-11) [43] explicitly avoids this binary framework, instead adopting a more nuanced model that includes onset-type specifiers (e.g., lifelong vs. acquired, generalised vs. situational) and a comprehensive list of potential etiological contributors—ranging from psychological and behavioural to medical and interpersonal factors [44]. This evolution reflects the increasing recognition of ED as a complex, multifactorial condition with overlapping biopsychosocial influences. Nonetheless, in clinical settings, the organic/psychogenic distinction remains a widely used and practical framework for diagnostic reasoning and treatment planning, particularly in surgical decision-making. Given the retrospective nature of our study and the need for categorical differentiation between patient groups, we used this classical terminology while acknowledging its limitations. We recognise that erectile dysfunction is a multifactorial condition and, while our classification was supported by clinical evaluation, psychosexual assessment and objective diagnostic tools, we recognise that such dichotomous labelling may oversimplify the complex interplay of aetiologies inherent in many ED cases. Another weakness is that the surgeries were performed by different surgeons and with more than one brand of prosthetic materials. This is a challenging issue in a large specialised teaching centre, due to the frequent modification of materials introduced by the device industry.

Larger prospective studies using ICD-11-based classifications are needed to confirm these findings and better understand the outcomes in psychogenic ED patients. Future research should also explore long-term satisfaction, partner perspectives and the potential role of psychological support in surgical decision-making.

## 5. Conclusions

Patients with psychogenic ED reported high satisfaction after penile prosthesis implantation, comparable to those with organic ED. Therefore, this intervention should not be withheld from patients with mainly psychogenic ED. However, a possible increase in complications in the psychogenic cohort highlights the need for careful consideration of surgical risks in this population, warranting further studies with a larger number of individuals.

## Figures and Tables

**Table 1:** Characteristics of the studied population that received a PPI.

	Total (N = 61)	Cases-PSY (N = 25)	Controls-ORG (N = 36)	*p*-Value *
Age (years)	63 (57–66)	61 (55–64)	64 (59–69)	0.068
Duration of ED (years)	6.0 (5.0–9.7)	6.0 (5.5–9.5)	5 (6)	0.206
Smoker (N, %)	13 (21%)	2 (8%)	11 (30.5%)	0.030
Hypertension (N, %)	35 (58%)	19 (76%)	16 (45%)	0.033
Diabetes mellitus (N, %)	24 (40%)	11 (44%)	13 (36%)	0.606
Dyslipidaemia (N, %)	41 (67%)	20 (80%)	20 (55%)	0.567
CAD (N, %)	15 (25%)	7 (28%)	8 (22%)	0.765

Cases-PSY: mainly psychogenic patients. Controls-ORG: patients with mainly organic ED. PPI: penile implant prosthesis. ED: Erectile Dysfunction; CAD: Coronary Artery Disease. All data are expressed as median (Interquartile Range between 25th–75th percentiles) or as frequency N (percentage). * *p*-value: cases vs. controls.

**Table 2:** Comparative analysis of the results of the QoLSPP questionnaire: Cases (PSY) vs. Controls (ORG).

Question	Cases-PSY (N = 25)	Controls-ORG (N = 36)	Total	*p*-Value *
1. How often do you evaluate if the penile prosthesis is adequate in relation to penetration and pleasure experienced?	3 (3)	4 (2)	4 (3)	0.197
2. How satisfied are you with the speed with which the prosthesis activates?	5 (1)	4.5 (2)	5 (1)	0.160
3. How satisfied are you with the duration of the prosthesis effects?	5 (0)	4.5 (2)	5 (1)	**0.039**
4. How often do you reach orgasm during sexual intercourse or masturbation?	5 (1)	5 (1.8)	5 (1)	0.565
5. How often have you had sexual activity using the prosthesis?	3 (2)	4 (3.5)	3 (2)	0.319
6. How would you evaluate the rigidity with the prosthesis compared to the rigidity before the prosthesis?	5 (1)	3 (2.7)	4 (2)	**0.011**
7. To what extent did the prosthesis meet your expectations?	5 (1)	4 (3)	5 (2)	**0.011**
8. How satisfied do you think your partner is with the functioning of your penile prosthesis?	5 (0)	4 (3.5)	5 (2)	**0.012**
9. To what extent has your penile prosthesis affected your and your partner’s well-being?	5 (1.5)	4 (2)	4 (2)	0.072
10. To what extent has your penile prosthesis affected your satisfaction in life?	5 (1)	4 (2)	4 (2)	**0.021**
11. To what extent has your penile prosthesis affected your general well-being?	5 (1)	4 (2)	5 (2)	0.176
12. To what extent has the prosthesis implant affected your feeling of being like other men?	5 (1)	5 (2.5)	5 (1.5)	0.158
13. How do you evaluate your desire to have sexual relations with your partner?	5 (1)	4 (2)	4 (2)	**0.013**
14. Do you feel more alive with your penile prosthesis?	5 (0)	4 (2.7)	5 (2)	**0.008**
15. How confident do you feel during sexual relations thanks to the penile prosthesis?	5 (0)	4 (2)	5 (1.5)	**0.015**
16. How do you feel about living the rest of your life in this condition?	5 (1)	5 (2)	5 (1)	0.305

PSY: mainly psychogenic ED patients. ORG: mainly organic ED patients. All data are expressed as Median (Interquartile Range between 25th–75th percentiles). * *p*-value: cases vs. controls.

## Data Availability

The data presented in this study are available on request from the corresponding author due to privacy, legal and ethical reasons.

## Abbreviations

The following abbreviations are used in this manuscript:

PPI	Penile prosthesis implantation
ED	Erectile dysfunction
MMAS	Massachusetts Male Aging Study
PCDU	Penile colour Doppler ultrasound
NPTR	Nocturnal penile tumescence and rigidity
PSY	Psychogenic ED
ORG	Organic ED
IIEF	International Index for Erectile Dysfunction

## References

[1] World Health Organization (WHO) World Health Organization Health Definition. https://www.who.int/teams/sexual-and-reproductive-health-and-research-(srh)/areas-of-work/sexual-health#:~:text=WHO%20defines%20sexual%20health%20as,of%20disease%2C%20dysfunction%20or%20infirmity.

[2] NIH Consensus Conference (1993). Impotence NIH Consensus Development Panel on Impotence. JAMA.

[3] Corona G., Petrone L., Mannucci E., Magini A., Lotti F., Ricca V., Chiarini V., Forti G., Maggi M. (2006). Assessment of the relational factor in male patients consulting for sexual dysfunction: The concept of couple sexual dysfunction. J. Androl..

[4] Feldman H., Goldstein I., Hatzichristou D., Krane R., McKinlay J. (1994). Impotence and its medical and psychosocial correlates: Results of the Massachusetts Male Aging Study. J. Urol..

[5] Gratzke C., Angulo J., Chitaley K., Dai Y.T., Kim N.N., Paick J.S., Simonsen U., Ückert S., Wespes E., Andersson K.E. (2010). Anatomy, physiology, and pathophysiology of erectile dysfunction. J. Sex. Med..

[6] Besiroglu H., Otunctemur A., Ozbek E. (2015). The Relationship Between Metabolic Syndrome, Its Components, and Erectile Dysfunction: A Systematic Review and a Meta-Analysis of Observational Studies. J. Sex. Med..

[7] Gandaglia G., Briganti A., Jackson G., Kloner R.A., Montorsi F., Montorsi P., Vlachopoulos C. (2014). A systematic review of the association between erectile dysfunction and cardiovascular disease. Eur. Urol..

[8] Nguyen H.M.T., Gabrielson A.T., Hellstrom W.J.G. (2017). Erectile Dysfunction in Young Men—A Review of the Prevalence and Risk Factors. Sex. Med. Rev..

[9] Caskurlu T., Tasci A.I., Resim S., Sahinkanat T., Ergenekon E. (2004). The etiology of erectile dysfunction and contributing factors in different age groups in Turkey. Int. J. Urol..

[10] Davis-joseph B., Tiefer L., Melman A. (1995). Accuracy of the initial history and physical examination to establish the etiology of erectile dysfunction. Urology.

[11] Hatzichristou D., Kirana P.S., Banner L., Althof S.E., Lonnee-Hoffmann R.A.M., Dennerstein L., Rosen R.C. (2016). Diagnosing Sexual Dysfunction in Men and Women: Sexual History Taking and the Role of Symptom Scales and Questionnaires. J. Sex. Med..

[12] Rosen R.C., Riley A., Wagner G., Osterloh I.H., Kirkpatrick J., Mishra A. (1997). The international index of erectile function (IIEF): A multidimensional scale for assessment of erectile dysfunction. Urology.

[13] Rosen R., Cappelleri J., Lipsky J., Pen Ä.B. (1999). Development and evaluation of an abridged, 5-item version of the International Index of Erectile Function (IIEF-5) as a diagnostic tool for erectile dysfunction. Int. J. Impot. Res..

[14] Ghanem H.M., Salonia A., Martin-Morales A. (2013). SOP: Physical Examination and Laboratory Testing for Men with Erectile Dysfunction. J. Sex. Med..

[15] Hatzichristou D.G., Hatzimouratidis K., Apostolidis A., Ioannidis E., Yannakoyorgos K., Kalinderis A. (1999). Erectile Dysfunction Hemodynamic Characterization of a Functional Erection Arterial and Corporeal Veno-Occlusive Function in Patients with a Positive Intracavernosal Injection Test. Eur. Urol..

[16] Sikka S.C., Hellstrom W.J.G., Brock G., Morales A.M. (2013). Standardization of Vascular Assessment of Erectile Dysfunction: Standard Operating Procedures for Duplex Ultrasound Sikka etal. SOP-Standardization of Vascular Assessment of ED. J. Sex. Med..

[17] Zou Z., Lin H., Zhang Y., Wang R. (2019). The Role of Nocturnal Penile Tumescence and Rigidity (NPTR) Monitoring in the Diagnosis of Psychogenic Erectile Dysfunction: A Review. Sex. Med. Rev..

[18] Gupta B.P., Murad M.H., Clifton M.M., Prokop L., Nehra A., Kopecky S.L. (2011). The effect of lifestyle modification and cardiovascular risk factor reduction on erectile dysfunction: A systematic review and meta-analysis. Arch. Intern. Med..

[19] Gerbild H., Larsen C.M., Graugaard C., Josefsson K.A. (2018). Physical Activity to Improve Erectile Function: A Systematic Review of Intervention Studies. Sex. Med..

[20] Collins C.E., Jensen M.E., Young M.D., Callister R., Plotnikoff R.C., Morgan P.J. (2013). Improvement in erectile function following weight loss in obese men: The SHED-IT randomized controlled trial. Obes. Res. Clin. Pract..

[21] Glina S., Sharlip I.D., Hellstrom W.J.G. (2013). Modifying Risk Factors to Prevent and Treat Erectile Dysfunction. J. Sex. Med..

[22] Aversa A., Bruzziches R., Vitale C., Marazzi G., Francomano D., Barbaro G., Spera G., MC Rosano G. (2007). Chronic sildenafil in men with diabetes and erectile dysfunction. Expert. Opin. Drug Metab. Toxicol..

[23] Porst H., Rajfer J., Casabé A., Feldman R., Ralph D., Vieiralves L.F., Esler A., Wolka A.M., Klise S.R. (2008). Long-term safety and efficacy of tadalafil 5 mg dosed once daily in men with erectile dysfunction. J. Sex. Med..

[24] Yuan J., Hoang A.N., Romero C.A., Lin H., Dai Y., Wang R. (2010). Vacuum therapy in erectile dysfunction-science and clinical evidence. Int. J. Impot. Res..

[25] Costa P., Potempa A.-J. (2012). Intraurethral Alprostadil for Erectile Dysfunction A Review of the Literature. Drugs.

[26] Eardley I., Donatucci C., Corbin J., El-Meliegy A., Hatzimouratidis K., McVary K., Munarriz R., Lee S.W. (2010). Pharmacotherapy for erectile dysfunction. J. Sex. Med..

[27] Sundaram C.P., Thomas W., Pryor L.E., Sidi A.A., Billups K., Pryor J.L. (1997). Long-Term Follow-Up of Patients Receiving Injection Therapy for Erectile Dysfunction. Urology.

[28] Lakin M.M., Montague D.K., VanderBrug Medendorp S., Tesar L., Schover L.R. (1990). Intracavernous injection therapy: Analysis of results and complications. J. Urol..

[29] Mulcahy J.J., Austoni E., Barada J.H., Choi H.K., Hellstrom W.J.G., Krishnamurti S., Moncada I., Shultheiss D., Sohn M., Wessells H. (2004). The Penile Implant for Erectile Dysfunction. J. Sex. Med..

[30] Mulhall J.P., Ahmed A., Branch J., Parker M. (2003). Serial assessment of efficacy and satisfaction profiles following penile prosthesis surgery. J. Urol..

[31] Vitarelli A., Divenuto L., Fortunato F., Falco A., Pagliarulo V., Antonini G., Gentile V., Sciarra A., Salciccia S., Sansalone S. (2013). Long term patient satisfaction and quality of life with AMS700CX inflatable penile prosthesis. Arch. Ital. Urol. Androl..

[32] Hatzichristou D.G., Hatzimouratidis K., Yannakoyorgos K., Dimitriadis G., Kalinderis A. (1998). Nocturnal Penile Tumescence and Rigidity Monitoring in Young Potent Volunteers: Reproducibility, Evaluation Criteria and the Effect of Sexual Intercourse. J. Urol..

[33] Wespes E., Amar E., Hatzichristou D., Hatzimouratidis K., Montorsi F., Pryor J., Vardi Y. (2006). EAU Guidelines on Erectile Dysfunction: An Update. Eur. Urol..

[34] Schlamowitz K.E., Beutler L.E., Scott F.B., Karacan I., Ware C. (1983). Reactions to the implantation of an inflatable penile prosthesis among psychogenically and organically impotent men. J. Urol..

[35] Barton G.J., Carlos E.C., Lentz A.C. (2019). Sexual Quality of Life and Satisfaction with Penile Prostheses. Sex. Med. Rev..

[36] Jorissen C., De Bruyna H., Baten E., Van Renterghem K. (2019). Clinical Outcome: Patient and Partner Satisfaction after Penile Implant Surgery. Curr. Urol..

[37] Akakpo W., Pineda M.A., Burnett A.L. (2017). Critical Analysis of Satisfaction Assessment After Penile Prosthesis Surgery. Sex. Med. Rev..

[38] Manfredi C., Fortier É., Faix A., Martínez-Salamanca J.I. (2021). Penile Implant Surgery Satisfaction Assessment. J. Sex. Med..

[39] Palasi S., Howell S., Green T.P., Kannady C., Slaughter K.B., Yang B., Panuganti S., Saavedra-Belaunde J.A., Clavell-Hernandez J., Wang R. (2022). Does knowing pre-operative penile length influence patient satisfaction post penile prosthesis implantation?. Int. J. Impot. Res..

[40] Caraceni E., Utizi L. (2014). A questionnaire for the evaluation of quality of life after penile prosthesis implant: Quality of life and sexuality with penile prosthesis (QoLSPP): To what extent does the implant affect the patient’s life?. J. Sex. Med..

[41] Dindo D., Demartines N., Clavien P.A. (2004). Classification of surgical complications: A new proposal with evaluation in a cohort of 6336 patients and results of a survey. Ann. Surg..

[42] Pineda M., Burnett A.L. (2016). Penile Prosthesis Infections—A Review of Risk Factors, Prevention, and Treatment. Sex. Med. Rev..

[43] World Health Organization (WHO) International Classification of Diseases, 11th Revision (ICD-11). https://www.who.int/standards/classifications/classification-of-diseases.

[44] Başar K. (2025). The Changes in ICD-11 Related to Sexual Health and Dysfunction and Their Implication for Clinical Practice. Turk. Psikiyatr. Derg..

